# Numerical instability of Hill-type muscle models

**DOI:** 10.1098/rsif.2022.0430

**Published:** 2023-02-01

**Authors:** Sang-Hoon Yeo, Jasper Verheul, Walter Herzog, Shinjiro Sueda

**Affiliations:** ^1^ School of Sport, Exercise & Rehabilitation Sciences, University of Birmingham, Birmingham, UK; ^2^ Cardiff School of Sport and Health Sciences, Cardiff Metropolitan University, Cardiff, UK; ^3^ Human Performance Laboratory, Faculty of Kinesiology, University of Calgary, Calgary, Alberta, Canada; ^4^ Department of Computer Science and Engineering, Texas A&M University, College Station, TX, USA

**Keywords:** muscle mechanics, musculoskeletal simulation, Hill-type muscle model, biomechanical models, numerical instability, computer graphics

## Abstract

Hill-type muscle models are highly preferred as phenomenological models for musculoskeletal simulation studies despite their introduction almost a century ago. The use of simple Hill-type models in simulations, instead of more recent cross-bridge models, is well justified since computationally ‘light-weight’—although less accurate—Hill-type models have great value for large-scale simulations. However, this article aims to invite discussion on numerical instability issues of Hill-type muscle models in simulation studies, which can lead to computational failures and, therefore, cannot be simply dismissed as an inevitable but acceptable consequence of simplification. We will first revisit the basic premises and assumptions on the force–length and force–velocity relationships that Hill-type models are based upon, and their often overlooked but major theoretical limitations. We will then use several simple conceptual simulation studies to discuss how these numerical instability issues can manifest as practical computational problems. Lastly, we will review how such numerical instability issues are dealt with, mostly in an ad hoc fashion, in two main areas of application: musculoskeletal biomechanics and computer animation.

## 1. Introduction

Simulating body movement using muscle contractions is a critical step towards understanding human movement. In recent decades, simulation techniques for musculoskeletal systems have made remarkable progress, and have been successfully used in many areas of application including sport and exercise sciences, ergonomics, musculoskeletal rehabilitation engineering, and computer animation. However, although there have been extensive studies and improvements in the geometrical representation of the musculoskeletal systems and their efficient numerical simulation, the actual model of the individual muscle mechanics has been used without seriously questioning its veracity and appropriateness. The purpose of this article is to revisit the fundamental issues related to the numerical instability of the predominant phenomenological model used in musculoskeletal simulation, the Hill-type muscle model (HMM).

Like all living organs, skeletal muscle is itself a biological system of immense complexity. As can be seen from existing cross-bridge models of muscle force generation [1], building a realistic molecular-level model of muscle force generation mechanism often accompanies a large number of model parameters and equations. However, those who build musculoskeletal simulation models face a dilemma that, although they want an accurate muscle mechanics model, they often have to put unnecessarily stringent limitations on the complexity of the individual muscle model for the sake of preventing their simulation from being computationally intractable or being severely overfitted. At the same time, it would not be desirable to build purely data-driven models based only on statistical curve-fitting without any supporting foundational theory of muscle contraction, which significantly limits the scope of application of the model to the one defined by the data used for fitting. This is where phenomenological models such as the HMM come into play; rather than focusing on describing the detailed molecular-level mechanism, phenomenological modelling prioritizes building a succinct model that can sufficiently explain the observed phenomena, i.e. a muscle's gross mechanical behaviour. However, unlike data-driven models, phenomenological modelling also emphasizes the connection to grounding theories and aims to build empirically valid and theoretically acceptable models, which therefore provides a better justification for extrapolating the model and a wider range of scenarios.

Due to the pragmatic view of phenomenological modelling described above, building a simple model comes at a price of lower accuracy and limited scope of empirical explanation. Therefore, users of phenomenological models have to accept the fact that such simple models can only provide overall predictions of the most typical behaviours of the system. For the same reason, it would be rather unreasonable to try to depreciate HMMs by simply pointing out their prediction errors in various mechanical scenarios since such complete predictive power can only be achieved by giving up computational efficiency. However, despite the general awareness of these limitations, this article attempts to draw attention to some issues of HMMs that are not just the inevitable cost of model simplification but are originating from a major theoretical contradiction of our current understanding of skeletal muscle contraction. More importantly, it will be highlighted that these issues lead to critical computational flaws in musculoskeletal simulation, which can seriously affect the stability and tractability—not to mention the accuracy—of the simulation.

In the following sections, we will first briefly revisit the history of the HMM and its major assumptions (§2). Then, we will question the feasibility of the HMM as a standard phenomenological model for muscle mechanics by revisiting the major theoretical loopholes of the model on its numerical stability, and related mechanical behaviours including eccentric contraction and history dependency (§3). In particular, we will provide simple and intuitive simulation case studies demonstrating what real problems and failures of simulation can be caused by the inherent numerical instability of the HMM. After that, we will discuss how these issues are currently addressed by ad hoc assumptions or adjustments in musculoskeletal biomechanics (§4) and computer animation (§5), where HMMs are widely used for simulations.

This article is primarily, but not exclusively, targeted at researchers in the musculoskeletal simulation community who are using HMM-based musculoskeletal simulators, either due to simplicity and convenience, or for the safety of using the so-called ‘textbook model’, but may not be very familiar with these fundamental issues. We also hope that researchers in muscle mechanics who have expert knowledge will find this article informative in understanding how these issues are recognized and dealt with at higher layers of research. To this end, this article, especially the earlier sections (§§2 and 3), is intended to provide a narrative review providing essential background and intuitive examples demonstrating the issues, rather than focusing on an exhaustive review covering all findings and models in muscle mechanics modelling. For more exhaustive reviews, we direct readers to excellent books [2–6] and reviews [7–9] published on this topic.

## 2. Foundations of Hill-type muscle model

Here we will first provide a short overview of some foundational assumptions and basic components of the HMM. Due to the nature of this paper, the scope of this article will not go any further than required for users of musculoskeletal simulation to understand the issues of the HMM. Note that the version of HMM focused on in this article is the one that is most widely used as it is implemented in predominant open-source or commercial musculoskeletal simulators (e.g. OpenSim, Anybody or MuJoCo), while there are other—normally much more sophisticated—versions of the HMM such as Virtual Muscle [10–12] or other variations that aimed to include more detailed contractile mechanisms [12–16]. Also, it is worthwhile to point out more recent efforts made toward muscle simulation models that are not purely phenomenological, by integrating detailed models of cross-bridge mechanics or motor unit recruitment, such as population-based models [12,17–19] or implementation of phosphate kinetics [15]. An overview can be found in reviews [7–9].

### 2.1. The main assumptions: state space of muscle mechanics

**Figure 1.**
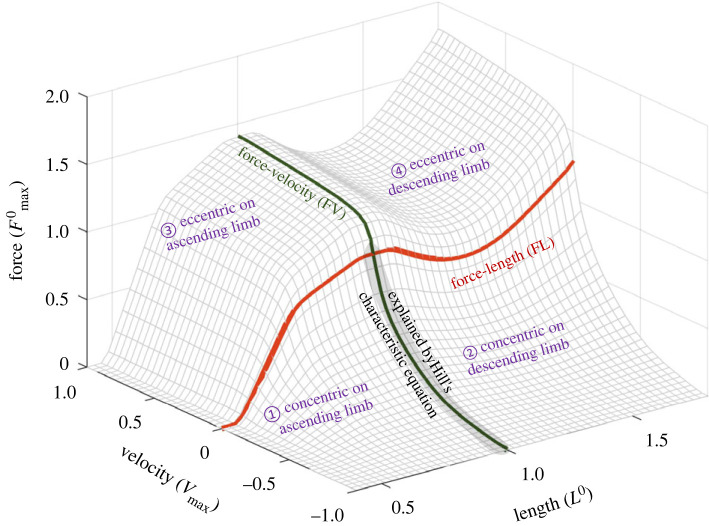
Force–length–velocity relationship assumed in HMMs and corresponding FL (red) and FV (green) curves. Given the length and velocity of a fully activated muscle, the surface predicts how much force it can produce. Based on FL and FV, four different contractile scenarios can be defined. The grey-shaded area is the only area on the surface predicted by Hill's equation.

Like any other system dynamics, a prerequisite for building a model of muscle mechanics is to define the state space, in which a point is mapped to a certain configuration (i.e. state) of the system. For a fully activated muscle, one main assumption of HMMs is that length and velocity, i.e. the rate of length change, define the state space of a muscle. In other words, the premise is that, for a fully activated muscle, knowing the current length and velocity is sufficient to determine the contractile force a fully activated muscle is generating. Based on this, the mechanics of a muscle can be visualized as a surface in the force–length–velocity space (figure 1), to which the dynamics of the muscle are constrained. If such a surface does exist, building a model of muscle mechanics would simply be the work of identifying that surface. The muscle contractile dynamics, i.e. how the length, velocity and force of muscle evolve over time, can be depicted as the trajectory of a point sliding on the surface.

Classic works of muscle mechanics have mainly focused on identifying two cardinal cross-section curves on this surface: force–length and force–velocity curves (red and green curves in figure 1; will be abbreviated to FL and FV hereafter). These curves dissect the surface along a fixed-length plane and the zero-velocity plane respectively. Identifying those two curves is an essential step towards understanding the shape of the entire surface, and most of the muscle parameters used for musculoskeletal simulation models are based on these two relationships. However, there are some important but often neglected caveats. First, these curves are assumed to be ‘non-dynamic’. In other words, it is not possible for a muscle's state to change along these curves, simply because the length cannot be changed while velocity is kept zero, and the length cannot stay constant when the velocity is nonzero. Second, even if the two major cross-sections of the surface are identified, how the surface is constructed from these two curves remains undetermined. Lastly but most importantly, it is not clear whether such a surface really exists. In fact, we do not yet have any valid answers to the question of whether this is the proper choice of state space (see §3.2 for further discussion). Keeping these precautions in mind, the sections below will be focused on the definitions and related issues of the FL and FV curves.

### 2.2. Hill's equation: the force–velocity curve

**Figure 2.**
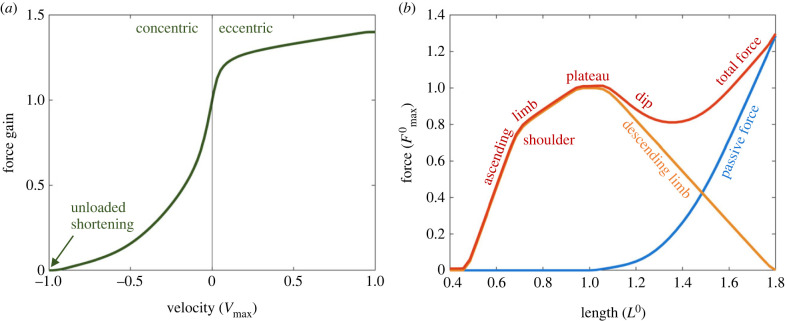
A typical FV and FL curve of HMMs, based on Millard *et al*. [21]. (*a*) FV combines concentric (negative velocity) and eccentric (positive velocity) regions, where the concentric part is based on Hill's equation. (*b*) Total FL (red) is a combination of active (orange) and passive (blue) FL curves. Names of different regions of the curve are based on the shape: ascending (with shoulder) and descending limbs are divided by the plateau formed around *L*^0^, the optimal length. The passive force that begins to develop after *L*^0^ cancels out the negative slope of the active FL, but often leaves a dip region where the negative slope still persists.

Modern models of muscle mechanics are grounded upon Hill's celebrated equation [20]. This equation suggests that the relationship between the contractile force of a muscle and its shortening velocity can be well modelled with the following hyperbolic equation:

2.1 (F+a)(−V+b) = c,

where *F* is contractile force, *V* is the velocity (i.e. the rate of length change; note that, by this definition, we consider concentric velocity to be negative, whereas many textbooks define concentric velocity as positive), and *a*, *b* and *c* are constants. This corresponds to the concentric region of the FV curve shown in figure 2*a*. An intuitive interpretation of this relationship is that the force produced by a muscle and its shortening velocity (−*V*) has a reciprocal relationship. That is, a muscle can operate either in high-force–low-velocity mode or in low-force–high-velocity mode, just like gears in a vehicle. The intercepts of this hyperbola with the force and the velocity axes determine the maximum isometric contractile force $F_{\max}^{0}$, the force produced by muscle in an isometric setup (i.e. *V* = 0), and the maximum shortening velocity −*V*_max_, the highest shortening velocity that muscle can achieve when *F* = 0. (For this reason, it is also called unloaded shortening velocity.) By replacing *c* with $F_{\max}^{0}$ or *V*_max_, the equation can be rewritten as

2.2 $$(F+a)(-V+b)=(F_{\max}^{0}+a)b=a(-V_{\max}+b).$$

If the force and the velocity are normalized with respect to $F_{\max}^{0}$ and *V*_max_, then by introducing a new constant $k=a/F_{\max}^{0}=b/V_{\max}$, the equation becomes

2.3 ( f+k)(−v+k)=k(−1+k),

and this leads to the normalized FV relationship shown in figure 2*a*:

2.4 $$f_{\;}^{v}(v)=\frac{k(v-1)}{k-\;v}.$$

Hill's equation accurately describes the FV relationship of the muscle, including skeletal [20], cardiac [22] and smooth [23] muscles. Notwithstanding its huge success, Hill's equation has important limitations that are well known, but are not adequately considered in the generalization of its use—the equation only describes the *concentric* (i.e. shortening) dynamics of the muscle at its *optimal length* (also interchangeably called the plateau length or rest length), the length at which the muscle's active isometric force is maximized. In Hill's original experiment, this relationship was obtained by first activating muscle isometrically (i.e. at a fixed length), releasing it while providing resistance through varying external loads, and measuring the *initial* shortening velocities [20]. For these experimental conditions, $F_{\max}^{0}$ will be obtained when the external load is just equal to the muscle contractile force. When an external load *F* is between zero and $F_{\max}^{0}$, the muscle will undergo concentric shortening, of which the initial velocity is predicted by Hill's equation. In addition, due to this specific experimental design, the captured dynamics are limited to the exact length where the muscle is initially activated, and that length is normally chosen to be the optimal length of the muscle, denoted as *L*^0^. Note that the ‘0’ in $F_{\max}^{0}$ stands for the force obtained at *L*^0^.

Looking again at the length–velocity–force surface shown in figure 1, the region of the contractile dynamics explained by Hill's equation is limited to the vicinity of the shortening part of the FV curve (grey shaded area in figure 1). Other regions on the surface, such as the lengthening, i.e. the eccentric dynamics or dynamics at lengths different from *L*^0^ are not the regions that Hill's equation is focused on. Despite the long history of the FV relationship, the question of whether and how Hill's equation can be generalized to other areas of contraction dynamics is still an open question. For instance, as will be discussed in the later sections, the eccentric dynamics of the muscle are known to be vastly different from what is predicted by Hill's equation, and the muscle force at lengths beyond *L*^0^ cannot be represented as a single surface in the length–velocity space.

### 2.3. Sliding filament models: the force–length curve

In modern muscle physiology, the sliding filament model (or more specifically the cross-bridge model) is considered to be the standard model of muscle mechanics. The sliding filament model proposes that the muscle force originates from a group of cross-bridges formed in the region where two myofilaments, actin (thin) and myosin (thick), overlap in the sarcomere. In their seminal paper, Gordon *et al.* [24] explain elegantly how the length-dependency of the contractile force, represented by ascending, plateau, and descending limb (orange curve in figure 2*b*), can be explained by a geometric relationship between two sliding filaments based on the assumption that the muscle's contractile force is proportional to the number of cross-bridges that can be formed between the actin and myosin filaments. Especially on the descending limb, the overlap between actin and myosin becomes smaller as muscle length increases, and muscle force decreases linearly with the length, resulting in the negative slope in the FL relationship. This active force is combined with passive muscle force, which typically develops at lengths beyond *L*^0^ (but with exceptions, e.g. cardiac muscles) and monotonically increases (blue curve in figure 2*b*). If passive and active forces are combined, the total FL relationship is obtained (red curve in figure 2*b*). Due to the negative slope of the descending limb of the active FL curve and the positive slope of the passive FL curve, the total FL curve often has a ‘dip’ region after the plateau where its slope becomes negative (figure 2*b*).

Due to its name, it would be tempting to assume that muscle elasticity—more precisely the Cauchy elasticity—is represented by the FL relationship. This means that, when a fully activated muscle changes its length, the force stays on the FL curve and the stiffness is defined by the first derivative of this curve. Indeed, this is how elasticity is defined in HMMs. However, an important issue here is that the experimental method used to obtain the FL relationship does not guarantee this assumption. When the elasticity of a string-like object—say a rubber band—is measured, the simplest way would be to change its length and see how the force changes accordingly. If the length changes quasi-statically (i.e. at a speed low enough not to generate any viscous or inertial force) the FL curve can be directly obtained. However, such methods are not feasible for active muscles since a muscle cannot remain fully activated while its length is slowly changing; long-lasting tetanic contractions induce fatigue and damage to the muscle. Instead, the FL relationship is obtained by interpolating a series of isometric forces obtained from individual muscle contractions. That is, (1) one activates a muscle at a fixed length, (2) measures how much force is produced, (3) deactivates and passively moves on to the next length and (4) repeats the same procedure. Although there are good reasons to do so, these interpolation-based methods do not guarantee that the interpolated curve represents the length-dependent dynamics of the muscle [2]. In fact, it will be discussed later that the length-dependent dynamics of an activated muscle are very different from the FL relationship (see §§3.2 and 3.3).

### 2.4. Hill-type muscle model (also known as Hill–Zajac model)

In muscle mechanics, Hill's muscle model was superseded by the cross-bridge model. However, as discussed earlier, simpler phenomenological models that capture the mechanical behaviour of the muscle with relatively few parameters are still preferred in large-scale musculoskeletal simulations, for reasons of computational efficiency and tractability. By combining Hill's equation of the velocity-dependency, and Huxley's model of the length-dependency, and adding passive elasticities and other architectural parameters (e.g. pennation angle), Zajac [25] proposed a full constitutive model of muscle mechanics, commonly referred to as the HMM. In this model, muscle is modelled as a system combining activation (α), maximum isometric force ($F_{\max}^{0}$), normalized active FL (*f^l^*), FV (*f^v^*) and passive FL (*f^p^*) relationships:

2.5 $$F(\alpha ,l,v)=\alpha \;F_{\max}^{0}\{\;f^{lv}(l,v)+f^{p}(l)\}=\alpha \;F_{\max}^{0}\;\{\;f^{l}(l)\;f^{v}(v)+f^{p}(l)\},$$

where *l* and *v* are normalized length and velocity with respect to *L*_0_ and *V*_max_, respectively. Activation level α is defined as a normalized property, i.e. *α* = 1 when fully activated. The multiplicative combination of *f_l_* and *f_v_* models the muscle's force–length–velocity dynamics, i.e. *f ^lv^* = *f ^l^f ^v^*, which represents the surface plotted in figure 1. By taking this form, the HMM assumes that the velocity-dependency works as a dimensionless ‘gain’ of the length-dependent force, which means that the isometric muscle force is determined by the current length; i.e. *f ^l^*(*l*) is either attenuated by shortening or amplified by lengthening. Among many possible ways to combine the FL and the FV relationships, this specific multiplicative form is supported by the important prediction of the cross-bridge model that the unloaded shortening velocity (−*V*_max_) is independent of muscle length, demonstrated experimentally by a classic study by Edman [26]. However, the actual experimental validation of whether the dynamic muscle force can really be predicted by this model when the length and the velocity change together is still lacking. Further discussions on the validity of this assumption and a comparison of other possible forms can be found in Yeo *et al*. [27].

Additionally, it is worth discussing another main assumption of the HMM related to the activation level α. By modelling α as a normalized gain, as shown in equation (2.5), that linearly scales the force of a fully activated muscle at the same length and velocity, HMM assumes that the possible interaction between the activation level and the force–length–velocity dynamics is minimal. However, studies on validating HMM in physiologically relevant ranges of muscle activation levels, which are normally much lower than full activation, consistently reported substantial force prediction errors of HMM either in FL [28–30], FV [31] or force–length–velocity [32–35] conditions. This suggests that a muscle's force–length–velocity dynamics could be highly variable depending on activation level, and so cannot be linearly scaled. Notwithstanding clear experimental evidence, linear scaling is typically used as the standard formulation of HMMs.

Lastly, it is important to point out that another oversimplification of HMM-based muscle simulations is that they treat muscles as pure force generators that run in an ‘open-loop’ mode, while real muscles work in a ‘closed-loop’ environment, coupled with afferent regulatory neural mechanisms involving muscle spindles and Golgi tendon organs. These neural regulations allow muscles to run in versatile modes, such as impedance regulator, energy absorber, or instant stabilizer—see Nishikawa *et al.* [36] for a review. In order to simulate these higher-level control behaviours, recent computational models that implemented physiologically realistic closed-loop models of neuro-muscular mechanics can be considered [18,19,37–40].

## 3. Computational issues of Hill-type muscle model

As will be discussed in the later sections, HMMs have been widely used in simulation studies in biomechanics (see §4) and computer animation (§5) as a dominant phenomenological model of muscle mechanics. However, HMMs inherit the major assumptions of FV and FL relationships discussed above, and this causes some fundamental computational issues in simulations, which either can seriously undermine the reliability of the simulation results, or can even cause the numerical simulations to diverge.

### 3.1. Instability

Simulations based on HMMs can cause muscles to become uncontrollable in some scenarios due to their numerical instability. (Note that by calling it ‘numerical instability’, we are using a control-theoretical definition of system stability, which is clearly different from a descriptive term ‘stability’ that is often used in studies in biomechanics to describe smoother or more physiologically relevant model responses. Our definition of numerical stability is rather a dichotomous concept, and therefore there can be no ‘more stable’ or ‘less stable’ model.) This issue of instability is not limited to HMMs but is a major theoretical contradiction of the cross-bridge paradigm, and there have been long-lasting debates in muscle mechanics research [8,41]. Rather than reiterating those arguments, this paper will focus on how the numerical instability will affect actual computational simulations in practice. As already discussed in §2.3, if a muscle's contractile force at a certain length (with zero or negligible viscous force) is assumed to be governed by the FL relationship, this predicts that the muscle exhibits negative stiffness when the muscle length is on the ‘dip’ region on its descending limb (figure 2*b*). In other words, the active muscle force decreases as the muscle is lengthening. Note that some muscles, e.g. gastrocnemius, do not have dip regions in their FL curves since their passive forces develop more stiffly from shorter lengths [2,5], but our simulation studies will focus exclusively on muscles that have dip regions.

How does this negative stiffness affect the numerical stability of muscle? Let us assume that a muscle is modelled as a serial chain of force-generating elements (sarcomeres or any basis elements depending on the scope of modelling) where the mechanics of each individual element is modelled as an HMM. If all elements constituting a serial chain have the same length, the forces generated by them will be all equal and therefore the chain will be in its static equilibrium. However, if the length of one element in the chain becomes slightly different from others, the force produced by that element will change due to the length dependency, and the system will deviate from its static equilibrium. The problem of negative stiffness comes into play here. Let us assume that serially connected HMM elements are all on the dip region of the descending limb and thus have negative stiffness. If one element in the chain becomes slightly longer (i.e. stretched) than the others, the stretched element will become weaker than the others due to the negative FL relationship of the descending limb. This weakened element will be stretched further (and therefore will become even weaker) by neighbouring elements that become stronger by shortening, and this will continue until the element is eventually torn apart or the passive force becomes sufficient to offset the loss in the active force. Since a small perturbation in the state (i.e. length) results in an unrestorable deviation of the system from its equilibrium, this chain of HMM elements can be considered an unstable system in the dip region.

**Figure 3.**
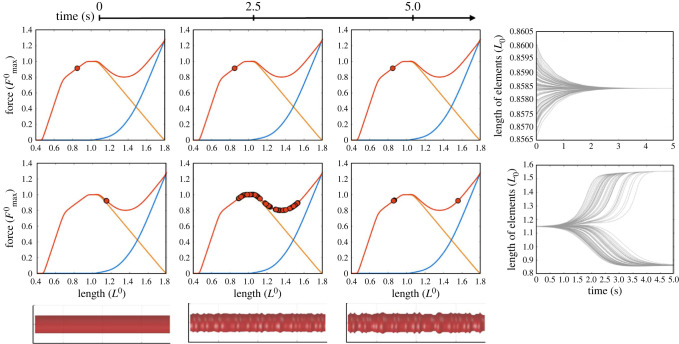
Simulations of one hundred serially connected HMM elements. In the first simulation (first row), the initial lengths of HMM elements were all set on the ascending limb (0.85*L*^0^) with less than 0.1% random non-uniformity across them. The first three columns are snapshots of the states of each element on the FL curve, taken at 0.0, 2.5 and 5.0 s, respectively, where filled red circles indicate lengths of HMM elements in these time points—note that this circle represents one hundred circles superimposed on each other. The second simulation (second row) had the same initial conditions as the first one, with the exception that the initial lengths were set on the descending limb (1.15*L*^0^). The snapshots and the length profiles clearly indicate a bifurcation of the HMM element lengths due to numerical instability. The rightmost column plots the simulated time–length profiles of all HMM elements for both conditions. Note that the top and bottom plots are at very different length scales. The visualizations at the bottom row are the corresponding snapshots of the volumetric deformation of the muscle based on the length profile simulated in the second simulation scenario. Each HMM element was modelled as an isovolumetric cylinder becoming thicker when shortened and thinner when lengthened.

Rigorous mathematical analyses of the numerical instability on the descending limb, e.g. eigenvalue analysis, can be found in theoretical studies [42,43], but here we provide a simpler and more intuitive example highlighting what would happen when simulating the dynamics of serially connected HMM elements. The model presented in figure 3 implements one hundred serially connected, fully activated (i.e. α = 1) muscle elements, each of which is modelled as the standard HMM adopted from Millard *et al*. [21] with a reasonable choice of model parameters: the rest length (*L*^0^) and the unloaded shortening velocity (−*V*_max_) of the whole chain were set as 30 cm (i.e. 0.3 cm for individual HMM element) and −10*L*^0^ s^−1^, and the mass and the maximum isometric force ($F_{\max}^{0}$) of the chain were set as 1 kg and 525 N, respectively. In this setup, the simulation was focused on the dynamics of the lengths of each element, when the total length of the chain was fixed. Two different fixed lengths were used: (1) when the muscle was at 85% of *L*^0^ (i.e. on the ascending limb) and (2) at 115% of *L*^0^ (i.e. on the dip region of the descending limb). At the beginning of the simulation, lengths of individual elements were all set to be equal, but a very small amount of random non-uniformity (within ±0.1% of the initial length) was added to each element.

Figure 3 summarizes the result of the simulation. On the ascending limb (figure 3, upper row), the initial non-uniformities vanish quickly and the whole muscle remains stable. However, these non-uniformities expand substantially on the descending limb (figure 3, second row), indicating that the model predicts numerical instability on the descending limb. Looking at the detailed behaviour of the HMM elements on the descending limb, it can be seen that they bifurcate and reach two different steady states: one group of elements settles at a length shorter than the initial length, while elements in the other group are stretched to a length beyond initial, where the reduced active force is compensated for by the increased passive force. If the simulation implements the three-dimensional shape deformation of the muscle based on isovolumetric contraction (i.e. the shape of each element is modelled as a cylinder and the volume of each cylinder is assumed to be constant throughout the simulation, making it thicker when shortened and thinner when lengthened), this unstable behaviour produces wrinkles as shown in figure 3 (bottom row). It has been speculated that this bifurcation could be a real phenomenon, called ‘sarcomere popping’, which has been reported to occur in real muscles [44]. However, this thinking has not been supported by recent experimental studies on single myofibrils [45–48], showing that the sarcomeres behave in a stable manner on the descending limb with no clearly observable bifurcation of sarcomere lengths. Further details of the previous and ongoing debates about the sarcomere popping hypothesis can be found in Minozzo & Lira [49].

The instability scenario presented above occurs when a muscle is modelled as serially connected HMM elements. This is a relatively rare choice in musculoskeletal simulation models unless the simulation incorporates volumetric muscles (see §5). However, the second scenario of numerical instability can occur in single-HMM-element musculoskeletal simulation models—when a pair of agonist and antagonist muscles are controlling a joint. Since two opposing muscles cannot be shortened or lengthened together in general, it would be reasonable to assume that, in some situations, one muscle is on its ascending limb and the other is on its descending limb. Then the total stiffness of the system is the combination of positive stiffness for the muscle on the ascending limb and negative stiffness for the muscle on the descending limb, and it is possible that the net effective stiffness of the system becomes negative. This will make the system work in an unstable manner similar to what has been observed in the simulation study above.

**Figure 4.**
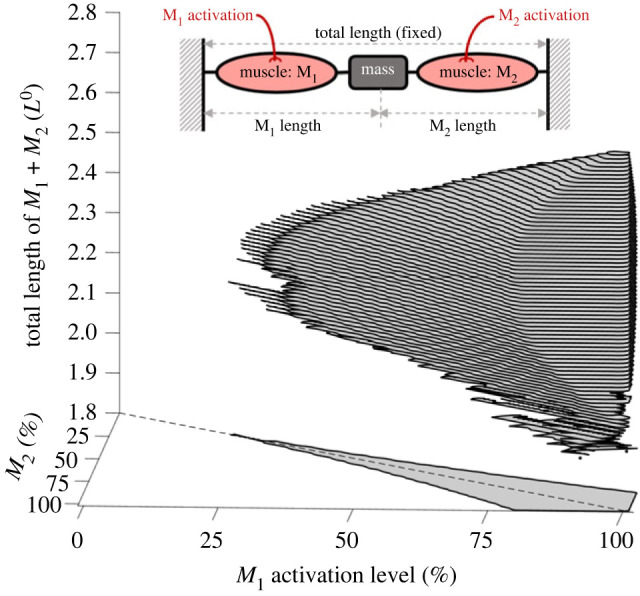
Prevalence of numerical instability in an agonist–antagonist system. The effective stiffness of the system was tested for different activation levels (*X* and *Y* axes) and total combined lengths (from 1.8*L*^0^ to 2.8*L*^0^). In this three-dimensional space, a group of points with negative effective stiffness is visualized as a grey volume, and its projection to the *X*–*Y* plane is drawn as a grey polygon. All the other points outside of the grey volume had a positive equilibrium.

To examine how frequently the unstable situation occurs, we provide an example of a simple simulation study. As a one-dimensional simplification of the agonist–antagonist muscle system, we consider two muscles, *M*_1_ and *M*_2_, that are connected to each other with a point mass (figure 4 inset). Let us assume that these two muscles have the same FL relationship with the same *L*^0^ but independent activation. The total length of the two muscles is fixed during a single simulation trial, but a set of simulation trials was run to cover different total combined lengths ranging from 1.8*L*^0^ to 2.7*L*^0^. Similarly, the activation levels of muscles were fixed during a single simulation trial but varied from 0% to 100% across different trials to cover the entire range of contractile scenarios. This resulted in a group of simulation trials in a three-dimensional parametric space of muscle activation levels (*X*-axis and *Y*-axis of figure 4) and total muscle length (*Z*-axis of figure 4) in which each point represents a single simulation trial. Within each trial, we first searched for static equilibria between two muscles, i.e. a pair of muscle lengths (whose sum equals the given total length) in which the two muscles generate equal forces. When an equilibrium point was found, we checked whether the net stiffness of the system was negative, i.e. if the equilibrium was unstable.

Figure 4 shows a group of points where the equilibrium was unstable. As shown, numerical instability was widespread: among all 1 030 301 points searched in space, 5.0% (51 654 points) of all solutions were unstable. This means that any random static simulation of an agonist–antagonist pair has a approximately 5% chance of being unstable. If the test region is reduced to a more physiologically meaningful range (i.e. ±10% of the rest length, or 1.8*L*^0^ to 2.2*L*^0^), 27.4% of all solutions are unstable. Numerical instability is more prevalent when muscles are co-activated equally (along the dashed diagonal line in the *XY* plane of figure 4), and when activation levels are high. This is rather intuitive since muscle forces should be similar to form an equilibrium, and the negative stiffness of the dip region on the descending limb becomes more prominent at high compared to low activation levels. Identifying regions of numerical instability becomes exponentially more complex for realistic simulation scenarios with more than two muscles, muscles with different resting lengths, and uni- and multi-articular muscles.

**Figure 5.**
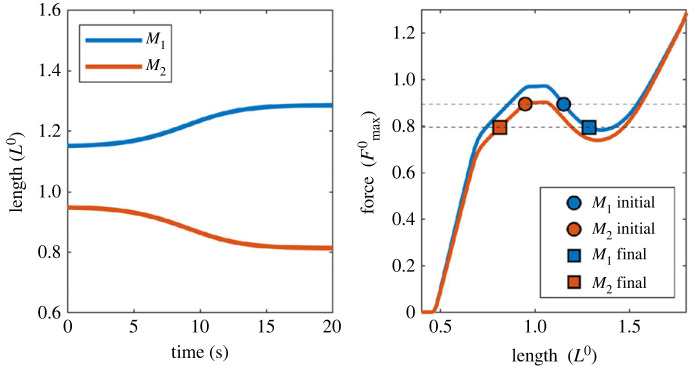
An example simulation of a two-muscle system with negative effective stiffness. Two muscles were activated at 91 and 98% respectively, and their FL relationships (normalized FL curve scaled by activation level) are plotted as blue and red curves in the left-hand panel. Initially, lengths of *M*_1_ and *M*_2_ muscles were set to 1.15*L*^0^ and 0.95*L*^0^, respectively, to form a force equilibrium at 0.894$F_{\max}^{0}$ (red and blue circles). When a small perturbation of 0.1% *L*^0^ was applied, the system deviated away from the initial condition and reached a stable equilibrium at 0.794$F_{\max}^{0}$ (red and blue squares).

Then, how will this agonist–antagonist system behave when the system is unstable? Figure 5 shows an exemplary simulation of unstable behaviour. Similar to the simulation with the chain of HMM elements, the standardized FL and FV relationships from Millard *et al*. [21] were used for each muscle with the same model parameters as used in the first simulation (i.e. $\left\{L^{0},V_{\max},\;F_{\max}^{0}\}\;=\;\{30\;\mathrm{cm},10L^{0}\;s^{-1},525\;N\right\}$) with a 1 kg mass between the muscles. When the total length of the two muscles was set to 2.1*L*^0^, it was found that muscles generate an equal amount of force and therefore reach a static equilibrium when one muscle is activated 91% at 0.95*L*^0^ and the other is activated at 98% at 1.15*L*^0^. For these initial conditions, the effective stiffness of the system was −0.572$F_{\max}^{0}$/*L*^0^ indicating that the system was unstable. When applying a small perturbation of 0.1% of *L*^0^, the two muscles diverged from the initial configuration, changed their length, and reached a stable equilibrium at 0.794$F_{\max}^{0}$ (figure 5).

The two simulation examples presented above, serially connected (figure 3) and agonist–antagonist pair (figures 4 and 5) of HMM elements, suggest that there are at least two different forms of numerical instability in HMM-based musculoskeletal simulations. These instabilities, and the resulting failure of the simulations, should not be considered simple prediction errors but should be recognized as a critical problem that jeopardizes the foundation of the HMM-based simulations. Realizing that musculoskeletal simulations with HMMs possess inherent numerical instability, the question arises as to why such numerical instability issues have not been reported in musculoskeletal simulation studies. We believe that there are several potential reasons.

Firstly, as previously mentioned, most of the muscle simulation models used in biomechanics do not include serially connected HMM elements but treat a muscle as a single element. This is an understandable choice since incorporating intra-muscular dynamics comes with a cost of a substantial increase in model complexity. (However, such simplification is generally not possible in computer animation, where simulating realistically deforming three-dimensional muscles is important. See §5 for an overview.) Although using a single muscle element is an easy solution for the sarcomere instability problem, it should be emphasized that this single-element simplification circumvents the problem of numerical instability only at a superficial level and leaves substantial errors in predicting the muscle dynamics on the descending limb of the FL relationship.

Secondly, the unstable behaviour seems to be mostly ‘inconspicuous’. The distribution of unstable equilibrium points shown in the second example suggests that numerical instability commonly occurs even in systems consisting of single-HMM muscles when both agonist and antagonist are highly co-activated. Given the fact that the level of coactivation of agonist–antagonist muscle stays low, e.g. no more than 20% of maximum force for leg muscles during locomotion [50], the real probability that musculoskeletal simulations undergo numerical instability could be lower than the 5% estimated in the above example. Moreover, even when the system becomes unstable, our example simulation in figure 5 indicates that the unstable behaviour tends to develop slowly due to the inertial and viscous forces, taking about five seconds until the system shows a noticeable change in muscle lengths, while many contractile scenarios may not include such long-lasting static co-contractions. Nevertheless, this only means that clearly visible failures are less likely to occur during the simulation, not that the reliability and accuracy of the simulation are unaffected.

Lastly, it is possible that the agonist–antagonist instability is inherently avoided by the way the musculoskeletal systems are designed. A classic study by Lieber & Fridén [51] on wrist extensors and flexors suggested that the stability of the multiple-muscle system of the wrist is maintained over almost the entire range of motion thanks to their very different FL properties. A study with a more detailed musculoskeletal system (e.g. [13]) on the conditions under which the stability of multiple-muscle systems is guaranteed, and whether these conditions are already reflected in the actual design principle of the biological musculoskeletal system, would be an interesting research direction. However, it will be shown in the next section that the stiffness of real muscles is vastly different from that predicted by the FL curve, and thus the instabilities introduced in musculoskeletal models may not exist in reality, independent of the detailed shapes of the FL relationships of individual muscles in a musculoskeletal system.

In the next sections, we will briefly review what kind of and how much error, compared to the mechanics of real muscles, is produced when simulating a single-element HMM, especially focusing on the mechanics of eccentric contraction on the descending limb.

### 3.2. History dependency: residual force enhancement

**Figure 6.**
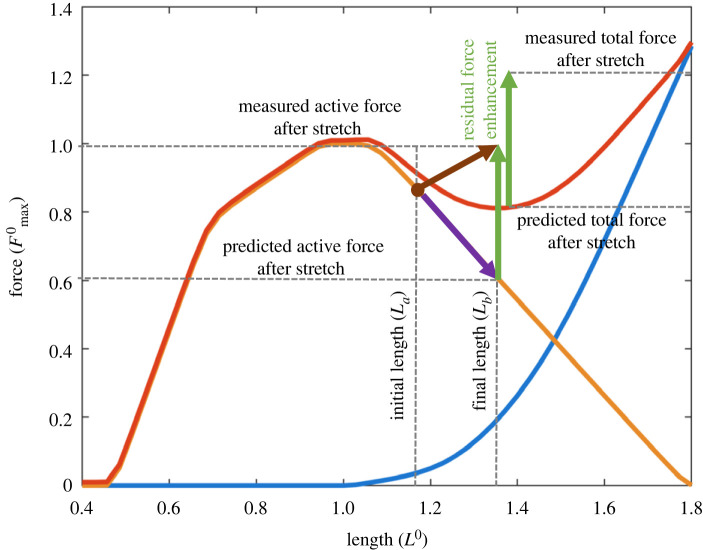
Schematic illustration of the residual force enhancement on the descending limb (the dip region) of the FL curve. The colour scheme of the FL curve is identical to the one previously used in figure 2*b*. A muscle is activated at *L*_a_, a length slightly beyond the plateau and therefore belongs to the dip region where negative stiffness is predicted by the HMM and stretched to a longer length *L*_b_. Whereas the HMM predicts a reduction of active muscle force (purple arrow) along the descending limb, the actual observed active force increases (brown arrow), indicating that the FL relationship does not stay on the FL curve anymore. The enhancement of active muscle force after the stretch (green arrow on the left) compared to the amount predicted by the FL curve is called residual force enhancement. In the actual experiments, the same amount of enhancement (green arrow on the right) will be observed in total force, under the assumption that passive force always follows the passive FL relationship (blue curve).

If real muscles are not unstable on the descending limb as has been perpetuated for more than half a century [52], what happens when muscles operate on their descending limb of the FL relationship? When a muscle is activated at some initial length *L*_a_ on the descending limb and then stretched to a longer length *L*_b_, the steady-state force achieved after the stretch substantially exceeds the isometric force that the muscle would achieve when it is activated at *L*_a_ as illustrated in figure 6. In other words, the muscle force does not stay on the FL curve and works as if it has a positive stiffness on the descending limb. This steady-state enhancement of muscle force on the descending limb induced by active stretch is called the ‘residual force enhancement’ (RFE). Research on RFE has a long history, starting with the early work of Abbott & Aubert [53], and has been acknowledged as an important property of muscles that cannot be explained by the sliding filament and cross-bridge model [54,55].

Importantly, the RFE provides strong evidence against the basic assumptions of HMM that are discussed above. First, the FL curve does not represent the length-dependency of the muscle's contractile force. As shown above, the dynamic length-dependent behaviour of active muscle force on the descending limb is very different from that predicted by the FL curve. RFE is also observed on the ascending limb [56] and the plateau [57] regions of the FL relationship, although RFE is typically not as pronounced as that measured on the descending limb. In addition, it is known that the opposite suppressive effect exists upon active shortening of a muscle, called residual force depression [53,58,59]. These findings do not only account for substantial errors in simulating eccentric contractions when history-dependent effects are neglected (see §3.3 below for details) but also lead to a major contradiction with the fundamental state-space assumption of HMM that the contractile force of a fully activated muscle is determined by its current length and velocity. In other words, the fact that muscles remain stable on the descending limb and that the dynamic muscle force does not follow the FL curve suggest that the muscle's contractile force exhibits history dependency; i.e. the muscle force also depends on the previous length (e.g. a length where it was first activated) and the rate of change in length. Implementing the history dependency not only improves the simulation accuracy of the mechanics on the descending limb but can also provide a complete solution for the instability issue of HMM. However, this can only be achieved by radically modifying the basic state-space assumptions of HMM by adding the contractile history as an extra state to the model. Alternative muscle models that incorporated the history dependency will be reviewed briefly in §4.

### 3.3. History dependency: dynamics of eccentric contractions

The issues of instability and the RFE suggest that the explanatory scope of HMM and the sliding filament model cannot cover well the muscle mechanics on the descending limb. In relation to that, another well-known but often neglected issue in phenomenological muscle modelling is the dynamics of active lengthening (i.e. eccentric contraction). While active shortening (i.e. concentric contraction) occurs when a muscle is shortening against an external force or inertia, eccentric contraction occurs when a muscle is resisting ongoing lengthening. Just as a car has both accelerators and brakes, the musculoskeletal system is controlled by a combination of concentric and eccentric contractions of individual muscles [60]. It is well known that the eccentric mechanics of muscle is very different from that of concentric contraction. Specifically, a common observation is that muscles tend to work as efficient ‘brakes’ when resisting lengthening against an external force. For a given metabolic energy, eccentrically contracting muscles can perform four times more mechanical work by spending less than half the amount of neural input in comparison to concentrically contracting muscles [61]. Despite some noticeable efforts made in modelling [11,12,62], this exceptional efficiency of resisting external tension has long been considered one of the mysteries of muscle contraction [41], but such neuro-mechanical advantages have been widely used in exercise science, including rehabilitation strategies, muscle strengthening, and injury prevention [63–66].

**Figure 7.**
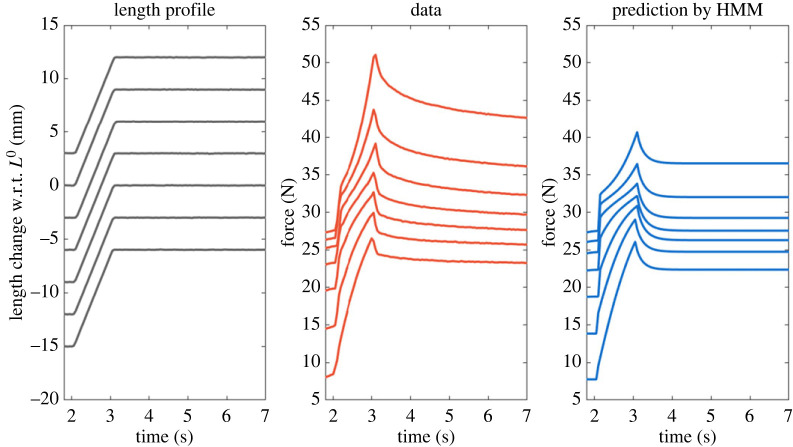
Comparison between measured and predicted dynamic force enhancement patterns. Muscle was activated and stretched with a constant velocity at seven different initial and final lengths, which covers the ascending limb, plateau, and descending limb (left) of the FL relationship. Experimentally recorded force profiles are plotted as red curves (middle) and predicted force profiles by a standardized HMM are plotted as blue curves (right).

Notwithstanding the abundance of empirical observations on the mechanical uniqueness of eccentric contraction, a rather surprising fact is that there is no model validated for the mechanics of eccentric contraction. As discussed earlier, Hill's equation only explains the dynamics of concentric contraction. The widely used HMMs assume the eccentric part of the FV relationship to be a smooth differentiable extrapolation of the concentric part of the FV curve (figure 2*a*), while in real muscles the contractile force rises much more rapidly during eccentric contractions [67,68] making the FV curve non-smooth around zero velocity. Using the extrapolated eccentric FV relationship, predictions using HMMs produce poor results when attempting to simulate the mechanics of eccentric contraction. Figure 7 shows eccentric force profiles of isokinetically stretched cat soleus muscle, data from Lee and colleagues [69], and the corresponding prediction by HMM. The active and passive FL curves used in the simulation are based on the data obtained from the same animal, and the FV curve was taken from one used in OpenSim [21]. As shown in the figure, HMM does a good job of predicting muscle rise patterns when the length of the muscle is less than *L*^0^. However, when the muscle length is greater than *L*^0^, i.e. on the descending limb, HMM substantially underestimates the force rise patterns during eccentric contractions. These observed force enhancements can be thought of as having originated from the passive viscosity, which is not modelled in HMM. However, the following steady-state enhancement of the force, i.e. RFE, indicates that the nature of the enhancement is not entirely due to the viscosity, but also due to a change in the elasticity, i.e. the history dependency.

### 3.4. Section summary

In summary, the HMM is the predominant phenomenological model of muscle mechanics but suffers issues with its numerical instability, especially when it comes to eccentric contractions or contractions at lengths beyond *L*^0^. It is true, of course, that all models are wrong, and the importance of a model should not be devalued just by pointing out its errors in different scenarios. Describing the mechanical behaviour of a highly complicated biological system with just a few parameters already premises, or even justifies, that there will be many aspects that cannot be predicted properly by the HMM. However, we put forward that the discussions and examples provided in this section suggest that the problems of numerical instability of the HMM cannot be excused by these general modelling practices, since such problems originate from faulty foundational assumptions underlying the HMM.

Firstly, we discussed that the FL curve in the HMM is regarded as the length-dependency that determines the muscle stiffness, which results in an interpretation that muscles can have negative stiffness on the descending limb of the FL relationship (dip region). This then leads to a major theoretical contradiction that a single muscle or a multiple muscle system can become mechanically unstable if they are modelled as a group of serially connected or opposing HMM elements, even though real muscles do not show this instability. The two simple simulation examples presented in this section have shown that the mechanical instability of HMMs can cause catastrophic failures for some contractile scenarios. We also speculated on reasons that could render these simulation failures inconspicuous in musculoskeletal simulations.

Secondly, the FV curve of the HMM is an extrapolation of Hill's equation that was originally derived to explain the relationship between initial velocity and force at the beginning of concentric contractions. Experimental evidence on the mechanics of eccentric contraction suggests that the HMM is limited in predicting RFE, and thus the state-space assumption of the HMM does not hold for real muscles. We also showed that the dynamic force enhancement during eccentric contractions preceding RFE deviates significantly from those of concentric contractions based on the cross-bridge model. We pointed out that the extension of the HMM to eccentric contractions was made without thorough efforts for validation or an estimation of possible error margins.

In the following sections, we will provide a brief overview of the existing solutions for issues related to stability, history dependency, and the mechanics of eccentric contractions of skeletal muscles, in two representative areas where musculoskeletal simulations are widely used: biomechanics (§4) and computer animation (§5).

## 4. The use of Hill-type muscle model for musculoskeletal simulations in biomechanics

Computational simulation models of the musculoskeletal system have been widely used in biomechanics to estimate and understand the mechanics of human movement that are difficult to measure *in vivo*. Musculoskeletal modelling and simulation platforms, such as OpenSim [70,71], Anybody [72,73], MSMS [74], MuJoCo [75] or demoa [76], combine models of muscle and tendon mechanics, neural inputs, skeletal and joint geometry and multibody dynamics, and use numerical methods to integrate the dynamics of the entire system. As with the intrinsic complexity of the motor system, musculoskeletal simulation models often exhibit a great amount of computational complexity, where movement is generated through hierarchical and parallel interactions of a large number of muscle–tendon units combined with multibody dynamics of the body segments. Although performing computations with these complex models is no longer a limiting factor of simulation studies thanks to the rapid growth of computational power, many users of musculoskeletal simulators, especially those without expertise in state-of-the-art muscle mechanics and biophysics, still prefer simpler conventional models due to their notational and computational convenience. For this reason, phenomenological models such as HMMs still remain as a preferred choice for musculoskeletal simulation studies compared to purely empirical or realistic but complex biophysical models.

Due to the multifaceted and hierarchical nature of musculoskeletal models, the overall simulation accuracy is determined by various factors involved at different levels of modelling. These include geometric and architectural representation of the muscle–tendon units, kinematics of bones and joints, and force sharing between multiple muscles. Since errors produced in lower-level components accumulate to higher levels, it can be reasonably assumed that errors arising from the most fundamental component of the model, i.e. the muscle mechanics model, have the highest impact on the accuracy of the whole simulation. Nevertheless, while most studies have focused on improving the performance of upper-level components, such as representations of muscle architecture or musculotendon geometry [21,70,77–81], or developing efficient algorithms for more accurate numerical simulation [82–85], the validity of the underlying muscle model in accurately predicting the behaviour of muscles for different mechanical scenarios has received substantially less attention. As discussed in the previous sections, the HMM suffers several limitations in explaining the history-dependency and the efficiency of eccentric contraction, which can lead to substantial errors in predicting the muscle's mechanical behaviour. Furthermore, these issues are fundamentally related to existing theoretical contradictions of the standard cross-bridge models on sarcomere instability. However, in studies on musculoskeletal simulations, these limitations are not addressed as much as other high-level problems, and the HMM is commonly accepted as the standard phenomenological model used for musculoskeletal simulations.

There are two possible reasons why the issues of HMM have not been highlighted as a major problem in musculoskeletal simulations. Firstly, as discussed in §3.1, computational problems associated with HMM-based simulations either are muted by simplification or are not readily apparent. Most musculoskeletal models used in biomechanical studies incorporate simple geometric representations of muscles where a serial connection of HMM elements is not implemented. In addition, instabilities in multiple muscle systems do not always become apparent during simulations of dynamic movement due to their slow progression. Secondly and arguably more importantly, the prediction errors produced by the muscle mechanics model can be diluted relatively easily during subject-specific adjustments of other musculoskeletal modelling parameters. It is well known that the performance of HMMs and the corresponding musculoskeletal simulation are highly sensitive to the selection of its muscle–tendon parameters, especially maximal isometric muscle force, optimal muscle fibre length and tendon slack length [86–89], which are also known to vary widely from individual to individual (e.g. [89]), and depend on sex, age, disease or physical activity level. Nevertheless, although substantial progress has been made to determine physiologically valid muscle parameters through advanced parametric sensitivity or topological analyses [17,90–92], the determination of these parameters in musculoskeletal modelling largely relies on rough estimations from the literature. For this reason, subject-specific tuning of those parameters is considered the common procedure for improving simulation accuracy. Adjustments are done by linearly scaling parameters by a scaling factor determined by individual anthropometrics, such as the bone or muscle length [71,93,94]. Alternatively, input parameters are adjusted to match those measured experimentally using dynamometry-based joint angle–moment relationships [87,95], ultrasound-based muscle–tendon interactions [96,97] and/or EMG-based muscle excitation [98,99]. These adjustments have been proven effective in improving the accuracy of simulations for individual subjects. However, it is not guaranteed that such adjustment procedures only compensate for the errors that arise from the choice of the parameter values, and not also from fundamental shortcomings of the model, or any other aspect of the simulation.

Meaningful efforts have been made to point out the severity of the HMM's prediction errors in a wide range of contractile scenarios, and to present an alternative phenomenological muscle model that can predict properties that are not explained by the HMM. For example, phenomenological muscle models have been developed to incorporate the history-dependent effects of muscle shortening or lengthening into the estimated muscle force [100–103]. Such models have been shown to reproduce history-dependent effects well for isolated muscles, they influence joint stability [100], lower-limb power output [104], muscle force magnitude and muscle coordination [105]. McGowan and colleagues demonstrated that force depression can reduce average crank power by as much as 40% during cycling [104] and that lengthening-induced force enhancement can increase the maximal force produced by the vastus lateralis by 22% during countermovement jumping [105]. However, it should be noted that such differences in model predictions can easily be compensated for by the tuning of other subject-specific parameters. For example, it is likely that the absence of history-dependent effects in the HMM can be compensated for by changes in muscle excitation for most submaximal activities [105]. This makes model performance comparisons between standard and history-dependent Hill models difficult.

Although the above-discussed studies highlight the importance of taking history-dependent effects into account for whole-body movement simulations, efforts on validating and utilizing such alternative models in the mainstream musculoskeletal simulation frameworks using, for example, standardized benchmark data [21,106–108] are still lacking. We speculate that the limited use of these alternative models is related to their practicability. These modified muscle models usually require extra tuning of model parameters and they may suffer from unrealistic discontinuities that are numerically difficult to handle [105]. These added complexities favour the use of computationally simple phenomenological models for large-scale simulations. Furthermore, the extra parameters and equations required to explain eccentric and history-dependent muscle properties are purely empirical since they are tailored to fit the experimental data, with no underlying fundamental model of muscle contraction. The absence of a strong connection to the underlying theories makes it questionable whether they could be called phenomenological models, not data-driven models.

In summary, despite awareness and efforts to improve the HMM's stability and performance in predicting eccentric and history-dependent mechanics, most musculoskeletal simulation studies in biomechanics use the standard HMM that is based on the problematic assumptions discussed in §2, and as a result, are not immune to the failures and prediction errors discussed in §3. We identified possible explanations as to why such fundamental issues have not emerged as major problems in biomechanical simulation studies. Aside from the modelling and numerical factors that effectively mask the aberrant behaviours of HMM-based systems, subject-specific tuning of musculoskeletal parameters can conceal errors arising from HMM's fundamental limitations.

## 5. The use of Hill-type muscle model for musculoskeletal simulations in computer animation

Musculoskeletal models have been used in computer animations because of the need to improve the rendering of human motion. Indeed, one of the holy grails of computer animation is to produce human animations that are indistinguishable from real human motion. To this end, both line-based muscle models, as well as geometrically complex, volume-based models that incorporate inter-sarcomere dynamics, have been used in computer animation.

### 5.1. Hill-type muscle model in line-based muscles

Line-based methods were developed by adding lines-of-action of muscles, just like those used in biomechanics (e.g. OpenSim). These muscles are assumed mass-less, taking the shortest path around obstacles between the origin and insertion [78].

Biomechanically motivated muscle models were first used in computer graphics for facial animation [109,110]. The origin and insertion of these muscles were based on anatomy, but the actual muscle model was a simple elastic spring. One of the first works in computer graphics to use the HMM was the work by Komura *et al*. [111–113]. They used inverse dynamics to first compute the required torques and then computed the muscle activations for the muscles to achieve these torques. Their use of proper muscle models allowed them to generate new types of animations that incorporated properties such as fatigue, which had not been possible with previous joint-torque-based approaches.

To generate plausible animations at a lower computational cost, many works in computer animation use simplified versions of the HMM. In these models, the FL relationship is often modelled as a rectified (i.e. negative value zeroed out) linear or exponential curve, and the FV relationship is either ignored or linearized in a similar way to the FL relationship. Lee & Terzopoulos [114] represented muscles as a combination of active and passive components, where the active component was modelled using rectified linear FL and FV curves, and the passive component was modelled as an exponential FL curve with linear damping. This simplified HMM was used to drive upper-body motion [115] and swimming [116]. Similarly, Sueda *et al*. [117] and Sachdeva *et al*. [118] used rectified linear functions for both active and passive FL relationships, with linear damping to approximate FV properties. These simplification procedures rule out the possibility of muscle models having negative stiffness and thus essentially prevent any computational issues arising from mechanical instability. However, they deviate greatly from the standard HMM and also from the realistic mechanical properties of muscles.

Recently, there has been a renewed interest in the use of full-fledged HMMs in computer animations. Wang *et al*. [119] showed that minimizing metabolic energy expenditure increases the visual realism of the resulting animations. Geijtenbeek *et al*. [120] used HMMs for a range of bipedal characters, including humans, animals and imaginary creatures. Similar to the subject-specific adjustments made in biomechanical simulations (see §4), they also optimized their animations for the placement and routing of muscle lines of action so that the total error based on speed, orientation and effort was minimized. Lee *et al*. [121] proposed a scalable biped controller that is able to solve for the activations of more than one hundred muscles, each of which is modelled as a parametrized HMM originally proposed in biomechanics [84]. Their animations include motions that simulate muscle pain, muscle tightness or joint dislocation. In their follow-up work, Lee *et al*. [121] used deep reinforcement learning to control more than three hundred HMM muscles for full-body motions. They were able to reproduce a wide range of motions, including muscle weakness, the use of a prosthesis and different pathological gaits. Despite the use of full-fledged HMMs, those authors did not mention the instabilities caused by HMMs. We speculate that this is because the movements in their simulations tended to have high velocities with little co-contractions. However, as discussed in §3, inherent instabilities still exist and may emerge during simulations involving static postures or slow movements.

### 5.2. Hill-type muscle model in volume-based muscles

Aside from the studies in biomechanics investigating volumetric mechanics of muscles—see [122] for a review—and the effect of distributed inertia [123], volume-based methods also have been widely used in computer animations in order to simulate visually realistic three-dimensional deformations of muscles. There are influential works that use non-biomechanics-based linear muscles—essentially changing the rest length of a linear spring model [109,124–126]—but these works will not be discussed in this paper. Among those that use biomechanically based muscle mechanics models, two subtypes have emerged. The first subtype uses a volumetric mesh, some edges of which are embedded with point-to-point muscle force generators, while the second subtype directly encodes the muscle as an anisotropic material model within the volumetric solid. We will discuss these two subtypes below.

The first subtype—those with embedded muscle force generators—was initially used in animation. Earlier works by Chen & Zeltzer [127] and Zhu *et al*. [128] introduced biomechanically based muscle mechanics models for computer animation. They used the finite-element method (FEM) with isoparametric brick elements, with the longitudinal edges of these elements acting as muscle force generators. Ng-Thow-Hing [129] used a similar approach to embed force generators based on the HMM inside a B-spline solid. Lemos *et al*. [130] developed a general FEM framework that could support any nonlinear material as the background isotropic material. Notably, they implemented the history-dependency (see §3.2) to deal with the negative slope of the FL curve by adding an additional linear term that keeps track of the length at which muscles were activated.

Around a decade after the introduction of the first subtype, the second subtype—those with anisotropic muscle material models—became popular. In the seminal work, Teran *et al*. [131] used a material model with strain energy that includes an anisotropic muscle potential term. This term was designed so that its derivative (i.e. force) matches the FL relationship from the HMM, approximated as piecewise quadratic and exponential functions. The FV relationship was not used in their model. The same muscle mechanics model was used in their follow-up work on large-scale simulations of skeletal muscles [132] as well as their research on facial muscles [133]. Recently, Lee *et al*. [134] simulated quasistatic volumetric muscles driven by per-element energy functions derived from an HMM. Most of these works use a piecewise quadratic active FL curve and an exponential passive FL curve that reduces the dip region of the descending limb compared to the FL curves used in typical biomechanical applications. However, it is not clear whether negative muscle stiffness and corresponding instability exist in their models since none of the papers includes any discussion of these issues. An interesting exception is a work by Fan *et al*. [135], which concedes that the muscle model is complex and not well understood, and instead allows the user to specify a time-dependent function to arbitrarily modify the desired shape of the muscle.

Regardless of which subtype of volume-based models is used, or whether there is a treatment to prevent instability within the muscle force generator, the forces produced by these volume-based models interfere with substantial background deformation forces; inertial, viscoelastic and/or volume-preserving forces built into the background isotropic material. This means that the total force produced by the entire muscle volume is a combination of muscle force and background deformation force, which may produce FL and FV relationships that are significantly different from those used in the embedded muscle force generator. In this setup, it is difficult to identify if the simulation of muscle volume suffers from the instability issue similar to the one with a serial chain of HMM elements (see §3.1), or if the background deformation force fortuitously works as a stabilizing force mitigating the instability of the inherent muscle model.

To summarize, graphic researchers have used HMMs to produce realistic-looking animations for both line-based and volume-based muscles. In most cases, simplifications were made to make the simulations scalable and robust, without rigorous verification. The fundamental instabilities discussed in this paper are not mentioned in these works (with some exceptions [118,135]), due to the fact that (1) the types of motion simulated tended to be fast with quick muscle velocities and little co-contractions or (2) the background deformation force unintentionally stabilized the simulations.

## 6. Conclusion

The HMM has been the most popular phenomenological model of muscle mechanics used in musculoskeletal simulation studies. In this paper, we revisited the HMM's basic structure with its underlying assumptions and discussed some fundamental problems of the HMM emanating from these assumptions. We provided conceptual case studies and experimental evidence suggesting that most musculoskeletal model simulations using standardized HMMs are at risk of failure and significant error due to inherent flaws. In the latter part of the paper, we discussed how such problems have been dealt with in HMM-based simulation studies in the areas of biomechanics and computer animation. Findings from this review of the literature suggest that most simulation models use the HMM with instability issues as they are, but these issues have not been recognized in general, possibly due to factors in modelling and parameter settings that can fortuitously mask errors or failures arising from HMMs. We discussed existing solutions to these issues, but most of the muscle models used for simulations are highly empirical, resulting in a significant increase in parametric complexity introduced to fit empirical data, or are made up for computational convenience in a highly simplistic and ad hoc manner rather than for producing accurate prediction of the mechanical properties of skeletal muscles.

With this paper, we do not attempt to offer an immediate solution to these problems. In fact, to the best of our knowledge, no alternative phenomenological model currently exists that has been adequately validated in terms of accuracy and usability. Instead, we hope that this paper will serve as a starting point for seeking better muscle models tailored for musculoskeletal simulation studies. As is often said, the first step in solving a problem is to recognize that there is one. Even if a suitable alternative model cannot be found—this is quite possible because there is no guarantee that we can accommodate the additional properties, such as the history dependency and eccentric mechanics, within a simplicity comparable to the HMM—we believe that it is important for anyone who is using the HMM in musculoskeletal simulation studies to understand the major issues and corresponding limitations.

What should be considered when attempting to find a better model that can be used for musculoskeletal simulations? Firstly, as repeatedly emphasized, efforts should be made to prioritize the balance between accuracy and computational convenience. Just as simple but inaccurate models are not desirable, models with physiological rigour but without fair consideration of their computational convenience will not be welcomed by the developers and users of musculoskeletal simulators. Secondly, because the existing issues of HMMs originate from the generalization of the FL relationship or Hill's equation with a limited effort of validation, developing a framework for more thorough validations is essential for the development of alternative models. In muscle mechanics, there have been continued efforts to develop alternative models of muscle mechanics, either based on an idea of including a new component, e.g. titin, to explain the mechanics of eccentric contraction [136–138], by considering intra-muscle dynamics [139,140], or by incorporating more elaborated mechano-physiological aspects [8,12–16]. Although translating them to validated and usable models would pose substantial research challenges, we believe that the effort to find a new model is well worth the work and is essential for more accurate simulation-based predictions of musculoskeletal mechanics in the future.

## Data Availability

This article has no additional data.
